# Bilateral vertebral artery dissection complicated by posterior circulation stroke in a young man: A case report

**DOI:** 10.1097/MD.0000000000022822

**Published:** 2020-10-30

**Authors:** Zhichao Li, Junni Liu, Xiang Wang, Xiaohui Liu, Qinjian Sun, Yifeng Du, Ling Yin

**Affiliations:** a Department of Oncology, The First Affiliated Hospital of Shandong First Medical University; b Department of Geriatric Cardiology, Shandong Provincial Hospital Affiliated to Shandong First Medical University, Shandong University; c Department of Neurology, Shandong Provincial Hospital Affiliated to Shandong First Medical University, Jinan, China.

**Keywords:** bilateral, case report, posterior circulation stroke, stroke management, vertebral artery dissection

## Abstract

**Introduction:**

Vertebral artery dissection (VAD) is a common cause of stroke in young and mid-aged adults without predisposing risk factors for vascular disease. It can be induced by a particular head or neck posture; its early signs often include headache and neck pain. Improved imaging techniques can be used to detect VAD, whose current treatment options are limited.

**Patient concerns:**

The patient presented with neck and shoulder pain for a week after sleeping against the wall with cervical proneness for 1 night. He had sudden headache, slurred speech, and left side weakness for 1.5 hours on admission.

**Diagnosis:**

The patient had VAD complicated by posterior circulation stroke.

**Interventions:**

Acute stroke was treated with intravenous thrombolytic therapy. Then, the patient was administered follow-up anticoagulants.

**Outcomes:**

The patient's condition improved after thrombolytic therapy. He recovered well, with no recurrence during a 4-year follow-up.

**Conclusion:**

VAD should be taken into consideration in differential diagnosis of posterior circulation stroke or transient ischemic attack in young patients. Intravenous thrombolytic therapy may be safe and effective for stroke-complicated cases. This case report demonstrates that expanded diagnostic protocol for acute ischemic stroke assures rapid and correct diagnosis.

## 1 Introduction

Vertebral artery dissection (VAD) is a common cause of stroke in young and mid-aged adults without predisposing risk factors for cerebrovascular disease.^[1]^ Headache and neck pain are often the early signs of VAD.^[2,3]^ Many improved imaging techniques can be used to detect VAD, including MRI (magnetic resonance imaging), MRA (magnetic resonance angiography), and DSA (digital subtracted angiography).^[4–6]^

The current treatment options are limited, with a marked shortage of controlled studies. Meanwhile, the safety and effectiveness of intravenous thrombolysis remain largely unstudied, with only case reports published.^[7–9]^ This work presents the case of a patient with acute ischemic stroke due to bilateral VAD, treated by intravenous thrombolysis and anticoagulants.

## 2 Case report

A 27-year-old male presented at the emergency department on September 10, 2015. The chief complaint was neck and shoulder pain for a week after sleeping against the wall with cervical proneness for 1 night. On admission, he had sudden headache, dizziness, nausea, slurred speech, a skewed mouth, and left side weakness, for 1.5 hours. He was 187 cm tall with about 45 kg in weight, and had disproportionately slender limbs, thin wrists and long fingers and toes. Neurologic examination revealed speech impairment and left-sided weakness (National Institute of Health stroke scale [NIHSS] = 10). He underwent mild reversion of the mitral valve many years ago. The patient denied any cocaine or amphetamine abuse. No other medical history was positive. Family history was not remarkable. Blood test indicated homocysteine at 17.4 μmol/L (normal ranges, 0–15 μmol/L), anti-myeloperoxidase antibody and protease 3 both at levels <2.00 IU/mL (normal ranges, 0–20 IU/mL), and rheumatoid factor levels at 9.19 IU/mL (normal ranges, 0–15.9 IU/mL). Other blood parameters were within the respective normal ranges. Abdominal ultrasound showed no abnormality.

**Figure 1:**
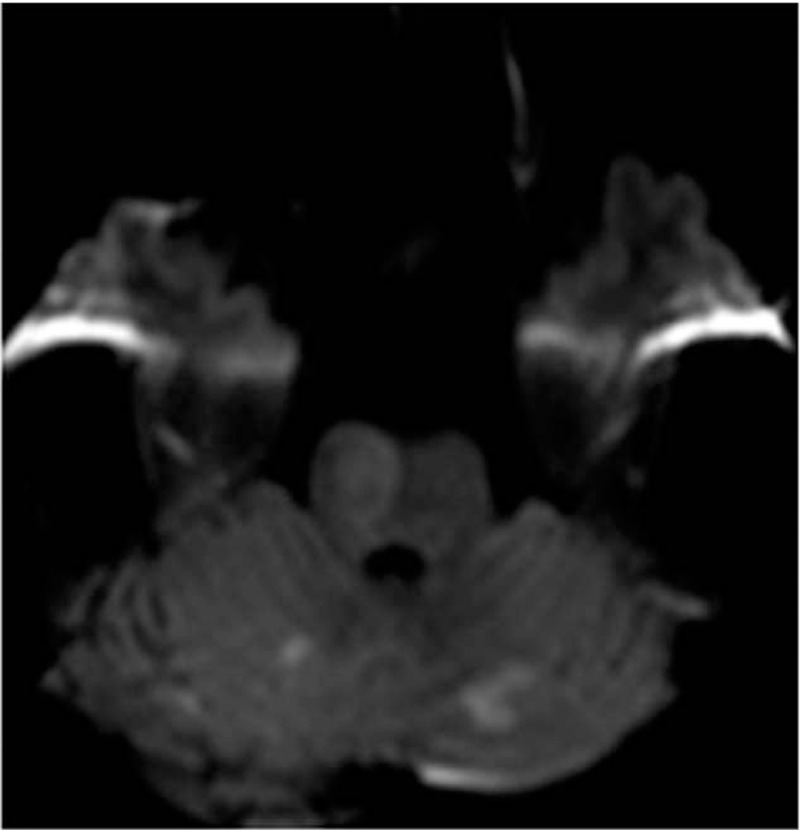
MR diffusion-weighted axial images 2.5 h after stroke. Infarct areas in the pons and both cerebellar hemispheres were evident.

**Figure 2:**
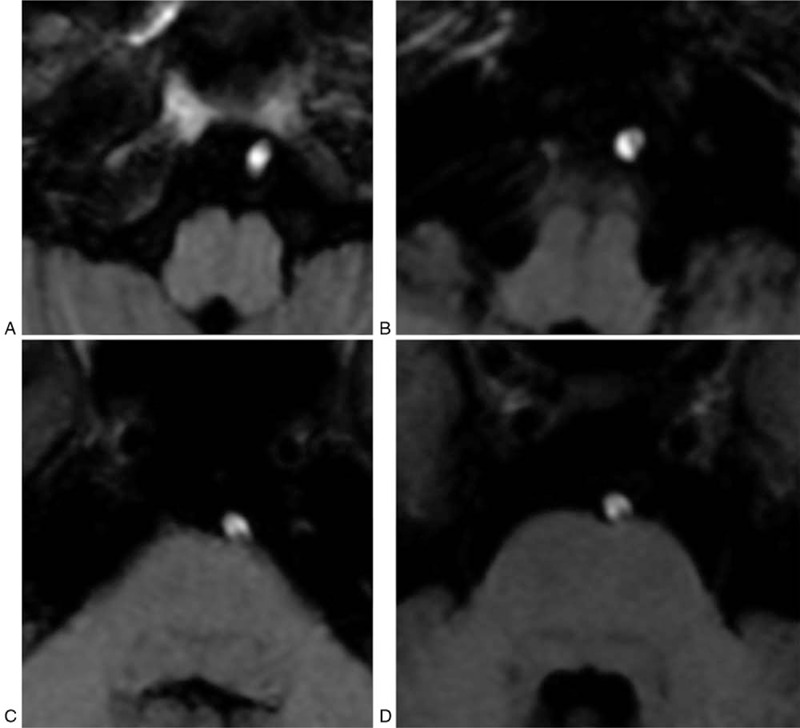
T1 weighted axial images. The left vertebral artery and basilar artery wall showed high intensity signals, indicating the presence of a hematoma. Loss of flow void signal in the left vertebral artery due to occlusion.

**Figure 3:**
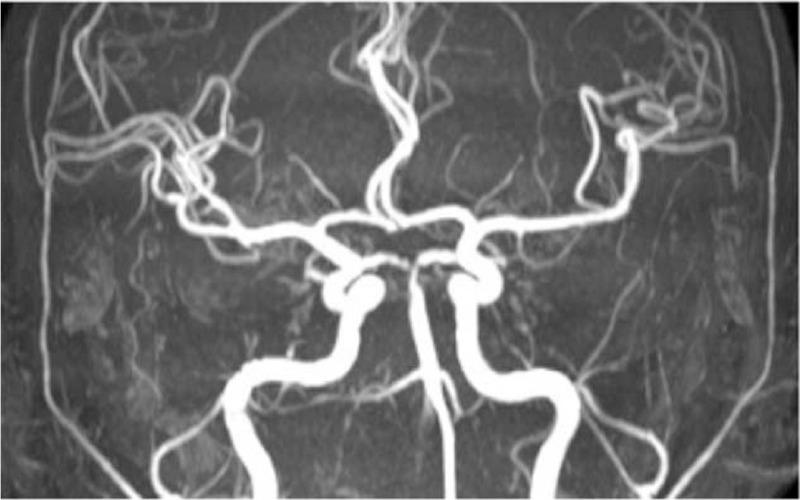
MRA results. The basilar artery and the origin of the posterior cerebral artery (P1 segment) were irregular and narrowed. MRA = magnetic resonance angiography.

**Figure 4:**
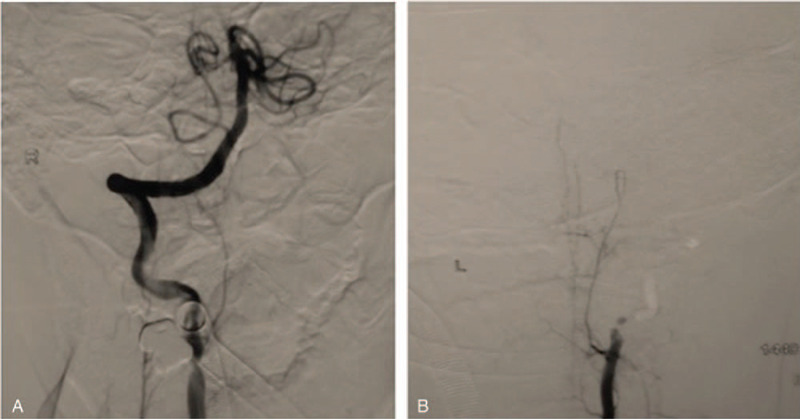
DSA data. Bilateral intraluminal filling defects in the V2 segment of vertebral arteries were evident. The left vertebral artery was occluded (B), while the right one was irregular and narrowed (A). DSA = digital subtracted angiography.

Head computed tomography performed 15 minutes upon admission was unremarkable. Then, the patient underwent MRI 1.5 hours after admission, and ischemic areas were detected in the pons and the cerebellum (Fig. 1). T1 weighted sequence showed high signals for the left vertebral artery (VA) and the basilar artery wall, indicating hematoma (Fig. 2). MRA showed irregular and narrowed basilar artery and origin of posterior cerebral artery (P1 segment) (Fig. 3). The patient was diagnosed with acute cerebellar and pons stroke. Within 4 hours of symptom onset, intravenous thrombolytic therapy was initiated. For treatment, recombinant tissue plasminogen activator at the standard dose of 0.9 mg/kg was administered (initial 10% of the total dose as a bolus over 1 minute, and the remaining dose over 60 minutes). Left side weakness symptoms were improved 30 minutes after the treatment. Emergency DSA confirmed the initial MRI and MRA findings, showing bilateral intraluminal filling defects in the V2 segment of VAs. The left VA was occluded, while the right one was irregular and narrowed (Fig. 4).

**Figure 5:**
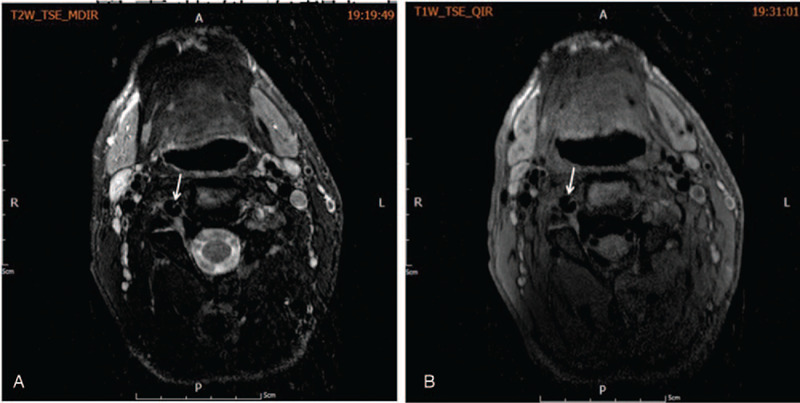
Magnetic resonance imaging (HR-MRI) findings. Irregular stenosis and intimal flap in the right VA (arrows) and occlusion of the left VA were observed. HR-MRI = high-resolution magnetic resonance imaging, VA = vertebral artery.

**Figure 6:**
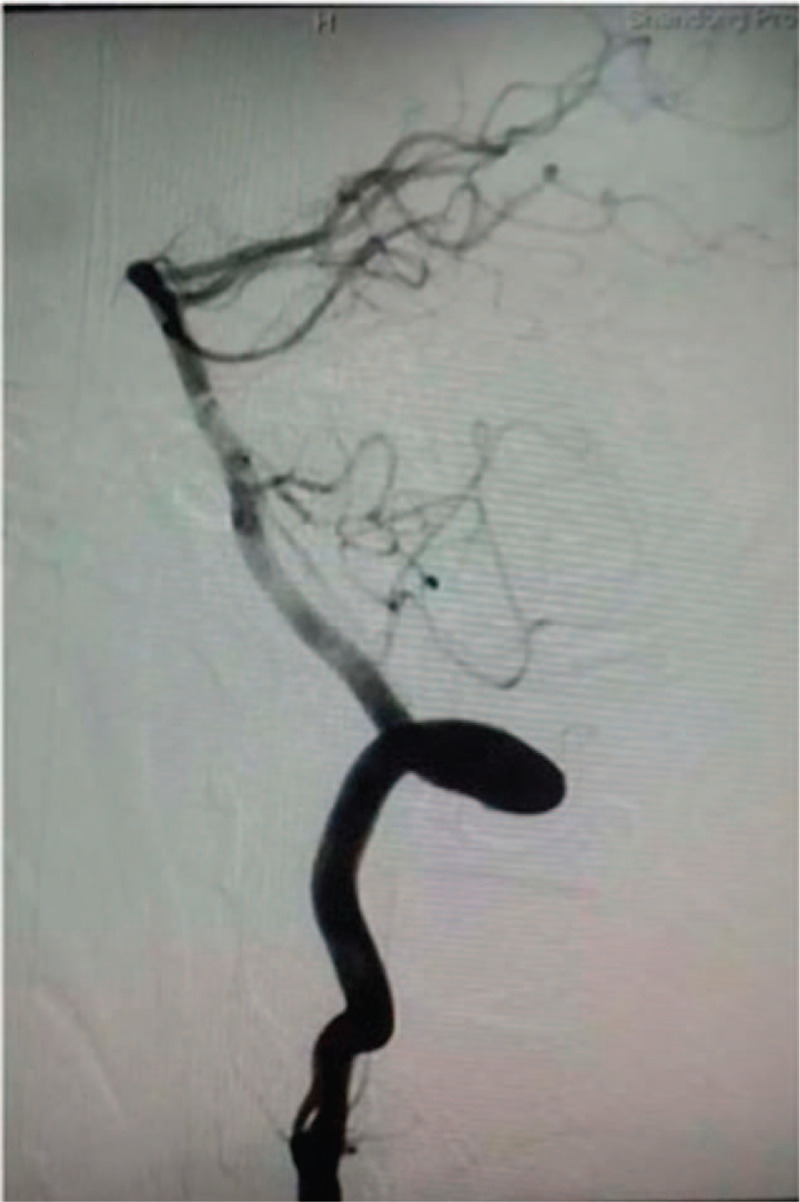
DSA examination 3 mo after stroke. The lumen of the right VA returned to normal. DSA = digital subtracted angiography, VA = vertebral artery.

Neurologic symptoms were improved significantly after the treatment (NIHSS = 6). Control computed tomography examination at 14 hours ruled out intracranial and cervical hemorrhage. High resolution-MRI revealed irregular stenosis and intimal flap in the right VA and left VA occlusion, which are dissection signs (Fig. 5). Anticoagulant therapy was started with low molecular weight heparin calcium (Fraxiparine, GlaxoSmithKline) (0.4 mL; hypodermic injection; q12h∗12 days). On the sixth day, oral Dabigatran was added at 110 mg bid for 9 days. Combination of both drugs was further administered for 7 days. After discharge, the patient was prescribed oral Dabigatran for 6 months at 110 and 160 mg mornings and evenings, respectively. The patient recovered well 3 days after the stroke (NIHSS = 0). Marfan syndrome was considered. FBN1 gene test was negative. In subsequent DSA examinations performed 3 months after stroke, the lumen of the right VA had almost returned to normal (Fig. 6) with left VA occlusion finally remaining. He recovered well and had no recurrence in the subsequent 4 years of follow-up.

This study was approved by the Ethics Standard Committee on Human Experimentation at Shandong Provincial Hospital Affiliated to Shandong First Medical University, Jinan, Shandong. Research has been conducted in accordance with the principles expressed in the declaration of Helsinki and its later amendments. The current patient provided informed consent to report this case.

## 3 Discussion

VAD is a rare and probably underdiagnosed cause of stoke, defined by the occurrence of a hematoma in the wall of an artery.^[10]^ It is an important cause of brain stem and cerebellar stroke, especially in the youth.^[1]^ Typical cases of VAD present with posterior circulation ischemic stroke in the brain stem (mostly Wallenberg syndrome), the thalamus, the temporo-occipital lobe, and the cerebellum after posterior neck pain or headache.^[2,3,11]^

VAD is usually caused by minor, rather apparently inoffensive precipitating events, including coughing, vomiting, chiropractic procedures, and blunt trauma; in addition, patients with connective tissue disorders are at increased risk.^[12]^ In the present case, the patient presented with neck and shoulder pain for a week after sleeping against the wall (phubbing), which is rarely observed.

Several imaging techniques are available for VAD diagnosis, including catheter-based DSA, contrast-enhanced computed tomography angiography, MRA, and ultrasound.^[5]^ The most common angiographic findings are irregular stenosis, occlusion, pseudoaneurysm, irregular dilatation, intimal flap, line-like symptom, and double lumen.^[13]^ DSA remains the gold standard for VAD diagnosis; however, MRA, computed tomography angiography, and DSA only clearly visualize the vessel lumen.^[14]^ Meanwhile, as in the current patient, hematoma of the arterial wall can be visualized on T1-weighted images with fat suppression as hyperintensity of the vessel wall, which is a frequently neglected hallmark for artery dissection. High-resolution-MRI can show not only arterial lumen abnormalities but also intramural and peri-arterial pathologies,^[15]^ and was equally applied in the current patient. It is worth noting that MRI and ultrasound findings in patients with VAD vary considerably, indicating that the management algorithm should encompass a multimodality approach taking into consideration the patient history, clinical neurological examinations, and imaging techniques.^[5]^

Treatment options for VAD are very limited, with a troubling shortage of controlled studies. Guidelines released in 2011^[15]^ state that antithrombotic treatment with either an anticoagulant or a platelet inhibitor for at least 3 to 6 months is reasonable for patients with extracranial VAD associated with ischemic stroke or transient ischemic attack. Indeed, anticoagulants and antiplatelet agents are equally valid for the secondary prophylaxis of stroke due to cervical artery dissection.^[16]^ Meanwhile, case series studies reported that intravenous recombinant tissue plasminogen activator may be effective and safe in patients with carotid artery dissection.^[1–3]^ Intervention or surgical operation should be considered in case of persistent pseudoaneurysm, or in patients who remain symptomatic due to thromboembolic events or show symptom progression despite anticoagulation treatment.^[17]^

Overall, the current case highlights the importance of considering VAD in the differential diagnosis of posterior circulation stroke or transient ischemic attack in young patients with a history of “phubbing.” Indeed, rapid and correct diagnosis of VAD is decisive in selecting proper treatment options and assuring good outcome. Intravenous thrombolytic therapy may be worth of recommendation in patients with VAD-related stroke.

## Acknowledgment

The authors appreciate the patient's cooperation sincerely.

## Author contributions

Ling Yin and Zhichao Li carried out the study, participated in collection, and drafted the manuscript. Junni Liu, Xiang Wang, Xiaohui Liu, Qinjian Sun, and Yifeng Du participated in the acquisition, analysis, and interpretation of the materials, and drafted the manuscript. All authors read and approved the final manuscript.

**Formal analysis:** Junni Liu, Xiang Wang, Ling Yin.

**Funding acquisition:** Junni Liu, Xiang Wang, Ling Yin.

**Investigation:** Zhichao Li, Xiaohui Liu.

**Methodology:** Qinjian Sun, Yifeng Du.

**Project administration:** Qinjian Sun, Yifeng Du.

**Resources:** Ling Yin.

**Software:** Xiaohui Liu.

**Writing – original draft:** Zhichao Li.

**Writing – review & editing:** Zhichao Li.
